# Impact of Early Life Antibiotic Exposure and Neonatal Hyperoxia on the Murine Microbiome and Lung Injury

**DOI:** 10.1038/s41598-019-51506-0

**Published:** 2019-10-18

**Authors:** Melissa H. Althouse, Christopher Stewart, Weiwu Jiang, Bhagavatula Moorthy, Krithika Lingappan

**Affiliations:** 1 Department of Pediatrics, Section of Neonatology, Texas Children’s Hospital, Baylor College of Medicine, Houston, Texas USA; 2 Alkek Center for Metagenomics and Microbiome Research, Department of Molecular Virology and Microbiology, Baylor College of Medicine, Houston, TX USA; 3 Institute of Cellular Medicine, Newcastle University, Newcastle, UK

**Keywords:** Respiration, Respiratory tract diseases

## Abstract

Cross talk between the intestinal microbiome and the lung and its role in lung health remains unknown. Perinatal exposure to antibiotics disrupts the neonatal microbiome and may have an impact on the preterm lung. We hypothesized that perinatal antibiotic exposure leads to long-term intestinal dysbiosis and increased alveolar simplification in a murine hyperoxia model. Pregnant C57BL/6 wild type dams and neonatal mice were treated with antibiotics before and/or immediately after delivery. Control mice received phosphate-buffered saline (PBS). Neonatal mice were exposed to 95% oxygen for 4 days or room air. Microbiome analysis was performed using 16S rRNA gene sequencing. Pulmonary alveolarization and vascularization were analyzed at postnatal day (PND) 21. Perinatal antibiotic exposure modified intestinal beta diversity but not alpha diversity in neonatal mice. Neonatal hyperoxia exposure altered intestinal beta diversity and relative abundance of commensal bacteria in antibiotic treated mice. Hyperoxia disrupted pulmonary alveolarization and vascularization at PND 21; however, there were no differences in the degree of lung injury in antibiotic treated mice compared to vehicle treated controls. Our study suggests that exposure to both hyperoxia and antibiotics early in life may cause long-term alterations in the intestinal microbiome, but intestinal dysbiosis may not significantly influence neonatal hyperoxic lung injury.

## Introduction

Bronchopulmonary dysplasia (BPD) is a chronic lung disease that remains one of the most common long-term complications of preterm birth. It results from a complex, multifactorial process that disrupts alveolar growth and pulmonary vascular development in the preterm infant lung^1^. Factors influencing this disruption include prematurity, hyperoxia, inflammation, prenatal and postnatal infections, mechanical ventilation, and growth restriction, amongst others. Although advances in care of the extremely low birth weight (ELBW) infant have improved survival rates, BPD incidence has plateaued. Antenatal steroids, non-invasive ventilation, volume -targeted ventilation, and surfactant administration have all greatly impacted the survival of the ELBW infant, however, there remains a need for additional novel strategies or therapies to prevent and treat BPD^2,3^.

There is increasing evidence that the microbiome plays a major role in maintaining a balanced immune response, establishing the gut barrier, and influencing disease development in the neonate^4,5^. The role of the intestinal microbiota in premature infants as well as the influence of prematurity on the development of the microbiome are emerging topics in research. Recent studies have suggested links between antibiotic associated intestinal dysbiosis in neonates and increased risk for preterm disease outcomes such as necrotizing enterocolitis^6–11^. Factors influencing the preterm gut microbiome include perinatal antibiotic treatment, use of non-human milk products, genetic factors, and prolonged hospitalization^4,12–14^. Overall, the preterm microbiome potentially impacts neonatal disease and long-term health outcomes of premature infants.

The cross talk between intestinal commensal organisms and the pulmonary system (the gut-lung axis) and its role in lung health and disease is actively being investigated. It is thought that intestinal dysbiosis may produce adverse pulmonary effects due to its potential to cause increased inflammation and impaired innate immunity^15^. Microbial dysbiosis has also been shown to play a role in chronic lung disorders such as asthma and cystic fibrosis^16^. The role of the gut microbiome in the pathogenesis of BPD and the underlying mechanisms have not yet been fully investigated. The objective of this study was to determine the effect of intestinal microbiome alterations on lung injury and repair after hyperoxia exposure in a murine model. We hypothesize that perinatal antibiotic-mediated intestinal dysbiosis increases BPD-like phenotype in a murine hyperoxia model.

## Methods

### Animals

We maintained active colonies of C57BL/6 wild type mice by breeding them in the animal facility at Texas Children’s Hospital’s Feigin Center. Time pregnant mice raised in our animal facility were used for the initial experiments (Ampicillin only) and timed pregnant mice obtained from Charles River Laboratories were used for the subsequent hyperoxia experiments. This study protocol and all animal experiments were approved by the Baylor College of Medicine IACUC and all experiments were performed in accordance with the IACUC guidelines and regulations.

### Mouse model of BPD

Mouse pups were allocated to either of two oxygen treatment groups, one group exposed to normoxia (room air at 21% O_2_) and the other group exposed to hyperoxia (95% O_2_), PND 4–8. Pups were returned to room air after hyperoxia exposure. We chose this timeframe for hyperoxia exposure since animals at this age are in the early alveolar stage of lung development. This model, which has previously been described, produces a distinct phenotype of arrest of pulmonary alveolarization and vascularization after hyperoxia exposure^17^. Plexiglas chambers (55 × 40 × 50 cm) were used to conduct hyperoxia experiments, and oxygen was delivered into the chambers through an oxygen blender to achieve a continuous level of 95% O_2_. Animals were examined every 12 hours for evidence of morbidity or mortality. Soda lime was placed in the chambers in order to remove excess CO_2_. Dams were rotated between room air and hyperoxia exposed litters every 24 hours to prevent oxygen toxicity in the dams. Dams exposed to antibiotics were only switched with other antibiotic exposed dams. Neonatal mice were euthanized on PND 8 (for microbiome analysis) and PND 21 after recovery in room air (for lung morphometry and microbiome analysis). The control group was kept in room air for the same duration of time (until PND 8 or PND 21).

### Antibiotic regimen

Pregnant dams were treated with ampicillin (100 mg/kg) via oral gavage prior to delivery from E18–20 to simulate prenatal antibiotic exposure. Pups were then treated with ampicillin (100 mg/kg) or PBS via intraperitoneal injection (i.p) for 3 days (PND 1–3). Control dams and control neonatal pups were treated with either PBS via oral gavage or PBS i.p. injections, respectively. Ampicillin is one of the most commonly used antibiotics for the empiric treatment of early onset sepsis in preterm neonates. Hence, we wanted to evaluate the effect of this antibiotic on the developing neonatal microbiome. In the second set of experiments with hyperoxia and room air, pregnant dams were treated with an antibiotic cocktail (ampicillin 5 mg/ml, gentamicin 0.2 mg/ml, vancomycin 0.8 mg/ml) combined and given at 0.2 ml per dose by gavage once a day from E16 - PND3 for a total of 7 days. Control dams received PBS via oral gavage at the same dose of 0.2 ml. In our second set of experiments, neonatal pups were not given antibiotics directly. The second set of experiments used an antibiotic cocktail to evaluate the effects of broad-spectrum maternal antibiotics on the neonatal microbiome as has been published in previous studies^18,19^.

### Lung histology and morphometry

Hyperoxia and room air exposed animals were anesthetized at PND 21 with 100 mg/kg sodium pentobarbital i.p., tracheas were cannulated, and lungs were inflation fixed with 4% paraformaldehyde (PFA) by endotracheal administration at 25 cm H_2_O pressure for 15 minutes. Next, the trachea was tied off and lungs removed and fixed overnight in 4% PFA at 4 °C followed by dehydration in graded alcohol and embedded in paraffin. Lung tissue sections were prepared and stained with hematoxylin-eosin and appropriate antibodies for immunohistochemistry. Alveolar development was assessed by the measurement of mean linear intercept (MLI) and radial alveolar counts (RAC) as previously described^17^.

### Pulmonary vascular development

To determine pulmonary vessel density, lung sections were stained for Von Willebrand Factor (vWF) (1:4000 dilution, abcam), an endothelial specific marker, using immunofluorescence techniques. Vessels were counted if the external diameter was less than 100 μm per high-power field. In the stained lung tissue section for each animal, we analyzed 15 random non-overlapping fields for microvessel number using the 40x microscope objective. The fields containing large airways or vessels were avoided.

### DNA extraction and 16S rRNA sequencing

Intestinal contents were obtained from neonatal mice as previously described, and both lung and intestinal contents were sent to the Alkek Center for Metagenomics and Microbiome Research at Baylor College of Medicine for 16S rRNA gene sequencing^20^. Methods were adapted from the NIH-Human Microbiome Project and the Earth Microbiome Project^21^. Bacterial DNA was extracted using the PowerMag Microbiome DNA isolation kit following the manufacturer’s instructions. The V4 region of the 16S rRNA gene was amplified by PCR and sequenced on the MiSeq platform (Illumina) using the 2 × 250 bp paired-end read protocol. The read pairs were demultiplexed and reads were merged using USEARCH v7.0.1090. Merging allowed zero mismatches and a minimum overlap of 50 bases, and merged reads were trimmed at the first base with a *q* ≤ 5. A quality filter was applied to the resulting merged reads and those containing above 0.5% expected errors were discarded. Sequences were stepwise clustered into OTUs at a similarity cut-off value of 97% using the UPARSE algorithm. Chimeras were removed using USEARCH v7.0.1090 and UCHIME v4.2. OTUs were mapped to a version of the SILVA Database containing only the 16S V4 region using USEARCH v7.0.1090.

### Statistical analysis

For morphometry analysis, GraphPad version 8 was used for the analysis of our data. Based on previous hyperoxia induced lung injury studies in our lab, it was estimated that 5 animals per group would provide 80% power at a significance level of 0.05 to detect a 25 μ increase (2 SD) in the MLI among hyperoxia exposed and room air controls. Data were expressed as mean ± SEM. Data were analyzed by two-way ANOVA to test for the independent effects of antibiotics and hyperoxia and to look for any interaction. Subsequent pairwise comparisons were completed using Tukey’s multiple-comparison testing to analyze the differences in treatments of hyperoxia and antibiotics in the control vs experimental groups. Significance was accepted as p < 0.05.

For the microbiome analysis, statistical significance for analysis of alpha diversity and taxonomic relative abundance between two groups used the Kruskal–Wallis test^22^. Differences in beta diversity (weighted Unifrac distance) were assessed using PERMANOVA. When comparing more than one measure, such as multiple measures of alpha diversity or for multiple taxonomic genera, *P*-values were adjusted for multiple comparisons with the false discovery rate (FDR) algorithm^23^.

## Results

### Early ampicillin alters the intestinal beta diversity and microbial composition

**Figure 1:**
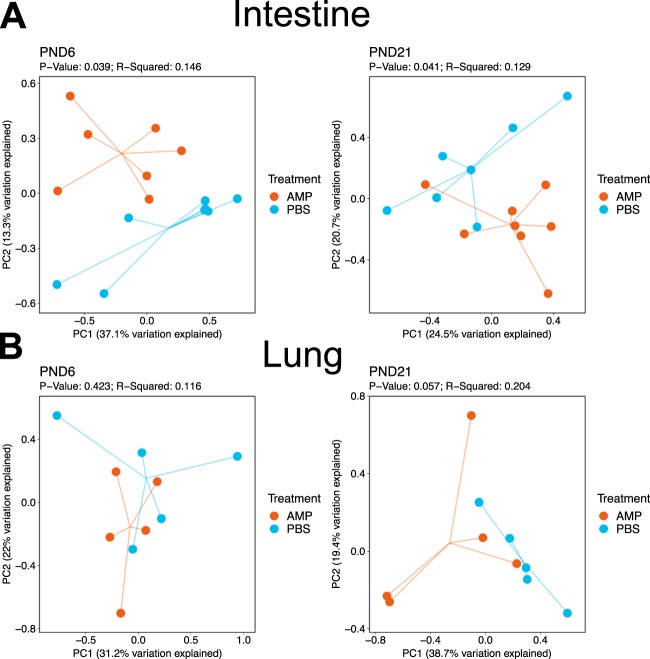
Early ampicillin exposure in neonatal mice alters intestinal microbial diversity. (**A**) Principal coordinate analysis plots represent intestinal beta diversity between ampicillin and PBS treated mice. Intestinal beta diversity was significantly different between ampicillin treated mice and controls at PND 6 (p = 0.039) and PND 21 (p = 0.041). (**B**) Lung beta diversity was not altered at either PND 6 (p = 0.42) or PND 21 (p = 0.057). Beta diversity was analyzed using both weighted and unweighted (Shown) UniFrac analysis. n = 5–8/group. AMP = ampicillin. PBS = phosphate buffered saline. PND = postnatal day.

**Figure 2:**
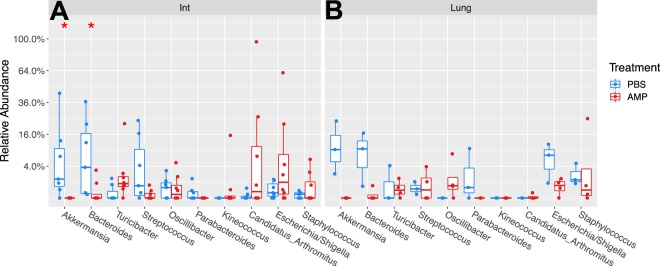
Ampicillin alters microbial composition in the intestine but not the lung at PND 21. (**A**) Taxa box plot A represents relative abundance at the genus level in the intestine at PND 21. *Bacteroides* and *Akkermansia* are decreased in Ampicillin treated mice compared to PBS controls. (**B**) Taxa box plot B represents relative abundance at the genus level in the lung at PND 21. There were no significant differences in microbial composition between ampicillin treated mice and controls in the lung. AMP = ampicillin. PBS = phosphate buffered saline. Int = intestine. n = 5–8/group. Significant differences between the ampicillin and PBS treated groups is represented by *P < 0.05.

Intestinal beta diversity was significantly different between ampicillin treated mice and controls at PND 6 (p = 0.039) and at PND 21 (p = 0.041) (Fig. 1A). However, Ampicillin did not alter lung beta diversity at either PND 6 (p = 0.42) or PND 21 (p = 0.057) (Fig. 1B). Ampicillin treated neonatal mice did not show significant differences in alpha diversity (Shannon Diversity or Observed OTUs) in the intestine or lung when compared to PBS treated controls at PND 6 or PND 21 (Supplemental Fig. 1). At PND 21, intestinal relative abundance of the genera *Bacteroides* and *Akkermansia* were significantly decreased in ampicillin treated mice when compared to controls (P < 0.05; Fig. 2A). However, we did not find any significant changes in relative abundance of commensal bacteria in the lung between ampicillin treated mice and controls (Fig. 2B).

### Neonatal antibiotic exposure with a three antibiotic regimen modifies intestinal beta diversity but not alpha diversity in neonatal mice

**Figure 3:**
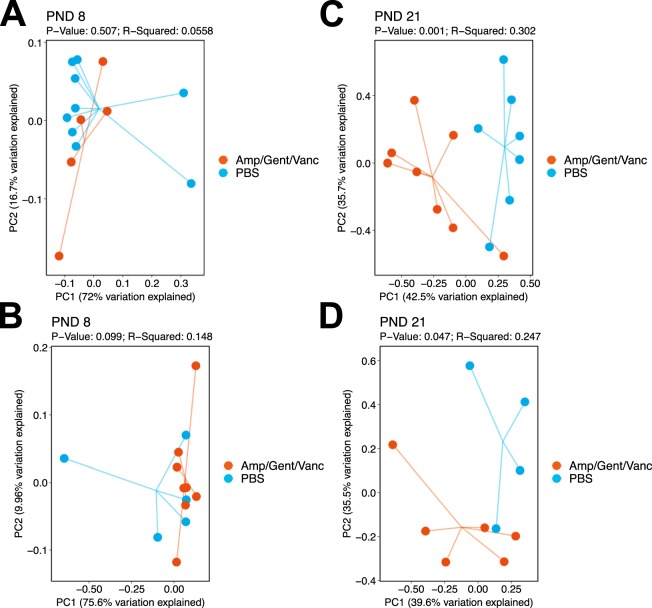
Perinatal antibiotic exposure with a three antibiotic regimen modifies intestinal beta diversity. Principal coordinate analysis plots represent intestinal beta diversity between three antibiotic (ampicillin, vancomycin, gentamicin) and PBS (control) treated mice stratified by oxygen exposure and timepoint. RA (**A**) and hyperoxia (**B**) groups exposed to antibiotics did not differ in beta diversity from controls at PND 8 (p = 0.51 and p = 0.099, respectively). At PND 21, intestinal beta diversity differed in both room air (p = 0.001; **C**) and hyperoxia (p = 0.047; **D**) exposed mice treated with antibiotics compared to PBS treated controls. n = 4–9/group. Amp/Gent/Vanc = Ampicillin, gentamicin, vancomycin antibiotic cocktail. PBS = phosphate buffered saline. PND = postnatal day.

At PND 8, no differences were observed in beta diversity in either room air (p = 0.51) (Fig. 3A) or hyperoxia exposed neonatal mice (p = 0.099) (Fig. 3B). Interestingly, there were significant differences in beta diversity at PND 21 with separation between antibiotic treated mice and controls in both the room air (p = 0.001) (Fig. 3C) and hyperoxia exposed groups (p = 0.047) (Fig. 3D). However, antibiotic treated neonatal mice did not show differences in alpha diversity measures using either Observed OTUs or Shannon Diversity when compared to PBS treated controls at either timepoint (Supplemental Fig. 2).

### Neonatal antibiotics modify relative abundance of intestinal commensal bacteria at PND 21 in neonatal mice

**Figure 4:**
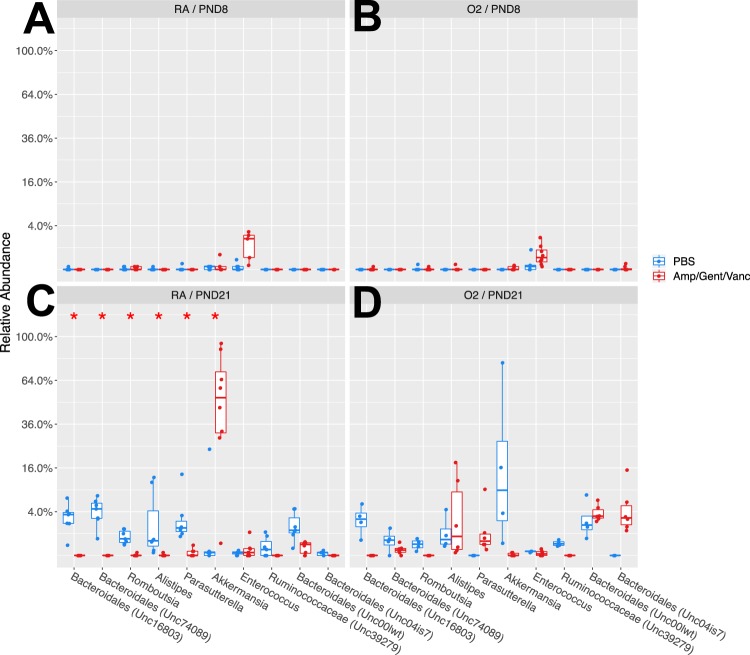
Perinatal antibiotics modify relative abundance of intestinal commensal bacteria at PND 21 in neonatal mice. At PND 8, there were no differences in relative abundance of genera between antibiotic exposed mice and PBS controls in either room air (**A**) or hyperoxia (**B**). At PND 21 in the room air group (**C**) there is a decrease in *Bacteroidales*, *Romboutsia*, and *Parasutterella* and an increase in *Akkermansia* seen in the antibiotic exposed mice when compared to controls (*p < 0.05). (**D**) There were no significant differences seen in relative abundance of intestinal commensal organisms in the hyperoxia group at PND 21 between antibiotic exposed and controls. n = 4–9/group. Amp/Gent/Vanc = Ampicillin, gentamicin, vancomycin antibiotic cocktail. PBS = phosphate buffered saline. PND = postnatal day. RA = Room Air, O_2_ = Hyperoxia.

There were no differences in relative abundance in commensal bacteria in the intestine seen at PND 8 between antibiotic exposed mice and controls in either room air or hyperoxia groups (Fig. 4A,B). However, at PND 21, in the room air group, there was a decrease in the relative abundance of genera *Bacteroidales*, *Romboutsia*, *Alistipes* and *Parasutterella* and an increase in *Akkermansia* in the three antibiotic exposed mice when compared to controls (p < 0.05) (Fig. 4C). There were no significant differences seen in the relative abundance of intestinal commensal bacteria in the hyperoxia group at PND 21 between antibiotic exposed mice and controls (Fig. 4D).

### Hyperoxia modifies intestinal beta diversity and relative abundance of commensal bacteria in antibiotic exposed mice

**Figure 5:**
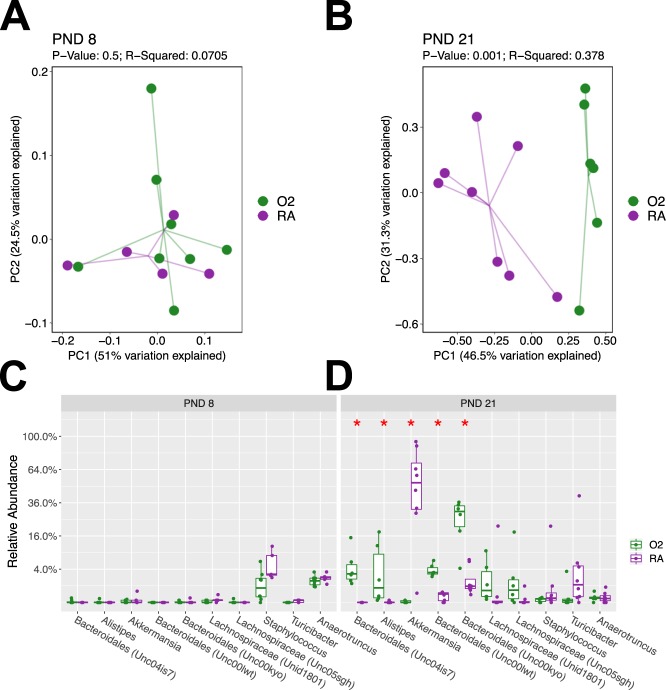
Hyperoxia modifies intestinal beta diversity and relative abundance of commensal bacteria in antibiotic exposed mice. (**A**) At PND 8, beta diversity was unchanged between room air and hyperoxia exposed mice (p = 0.5). (**B**) At PND 21, mice exposed to hyperoxia and antibiotics develop a distinctly different microbiota than those exposed to room air and antibiotics and beta diversity was statistically significant (p = 0.001). (**C**) No differences in relative abundance of different taxa were seen on PND 8. (**D**) At PND 21, there were significant increases in the genera *Bacteroidales* and *Alistipes* and a decrease in *Akkermansia* in the hyperoxia-exposed mice compared to room air. Analyzed using weighted UniFrac analysis. N = 5–8/group. PND = postnatal day. RA = room air. O_2_ = Hyperoxia.

Next, we wanted to elucidate if within the antibiotic exposed mice, hyperoxia had an early or delayed effect on the gut microbiome. We did not find any differences in beta diversity (p = 0.5) or relative abundance (p > 0.05) at PND 8 between the hyperoxia and room air groups among mice exposed to antibiotics (Fig. 5A,C). At PND 21, antibiotic exposed mice subjected to hyperoxia developed a distinctly different microbiome than those exposed to room air (p = 0.001) (Fig. 5B). There were significant increases in the relative abundance of *Bacteroidales and Alistipes* at PND 21 in the hyperoxia exposed mice compared to room air controls, however, there was a decrease in the relative abundance of *Akkermansia* (Fig. 5D). In the PBS treated controls, we did not observe any significant alterations in beta diversity or microbial composition between room air and hyperoxia exposed mice.

### Hyperoxia reduces pulmonary alveolarization and vascularization but the addition of antibiotics does not worsen lung injury phenotype

**Figure 6:**
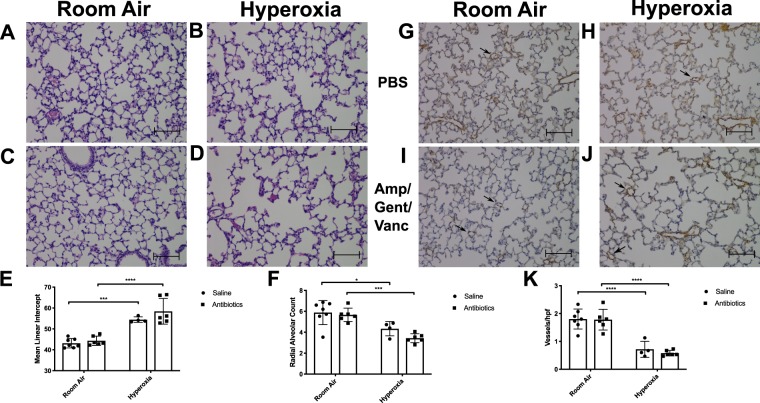
Hyperoxia reduces pulmonary alveolarization and vascularization but the addition of antibiotics does not worsen lung injury phenotype. (**A**–**D**) Representative lung sections with H&E at 20x magnification from neonatal mice exposed to RA or hyperoxia in addition to PBS or antibiotics. Mice exposed to hyperoxia (**B**,**D**) have decreased alveolarization. Mean linear intercept (**E**) was increased and radial alveolar count (**F**) was decreased in hyperoxia exposed mice. (**G**–**J**) Representative lung sections shown at 20x magnification subjected to immunohistochemistry for Von Willebrand factor. (**K**) Angiogenesis was assessed via microvessel count per high-powered field. Mice exposed to hyperoxia displayed a decreased vessel count/ high powered field when compared to controls. Scale bars represent 100 microns. (n = 4–7/group; Mean ± SEM). Data analyzed by 2-way ANOVA. Significant differences shown by ****P < 0.0001 and ***P < 0.001 and *P < 0.05. hpf = high powered field. PBS = phosphate buffered saline. Amp/Gent/Vanc = ampicillin, gentamicin, vancomycin antibiotic cocktail.

As described in the methods section, neonatal mice were exposed to room air or 95% oxygen for 4 days (PND 4–8) and then allowed to recover in room air until PND 21. Pulmonary alveolarization was determined using mean linear intercept (MLI) and radial alveolar count (RAC). Hyperoxia significantly reduces pulmonary alveolarization (p < 0.0001) which can be visualized in the representative lung fields at 20x (Fig. 6B,D) when compared to the room air controls (Fig. 6A,C). There was a significant increase in the MLI in hyperoxia + antibiotics exposed mice compared to room air + antibiotics (p < 0.0001). This was also seen in hyperoxia + PBS exposed mice when compared to room air + PBS exposed (p = 0.01). The MLI was not significantly different when comparing hyperoxia exposed mice with three antibiotic exposure to those without antibiotic exposure (p = 0.11) (Fig. 6E). RAC was significantly decreased in hyperoxia exposed mice compared to room air, both in antibiotic exposed mice (p = 0.0007) and PBS controls (p = 0.03). There were no significant differences seen in RAC between hyperoxia + antibiotic exposed mice and hyperoxia + PBS exposed mice (p = 0.33) (Fig. 6F).

Hyperoxia also causes an arrest in lung vascular development. To determine degree of angiogenesis, we quantified vessel number using immunohistochemistry for vWF as described in the methods section. Hyperoxia significantly decreased vessel development in the lung in both antibiotic and PBS exposed mice (p < 0.0001) which can be visualized in the representative lung fields at 20x (Fig. 6H,J) when compared to room air controls (Fig. 6G,I). There were no significant differences seen in the degree of decreased lung vascular development between hyperoxia + antibiotic exposed and hyperoxia + PBS exposed mice (p = 0.88) (Fig. 6K).

## Discussion

Although there has been an increase in survival of extremely premature infants over time, the incidence of BPD remains stagnant despite advances in care^1^. BPD has a multifactorial pathogenesis and identifying different mechanisms that contribute to its development is critical to developing therapeutic strategies that may prevent or ameliorate the severity of this disease. Our study sought to investigate how alterations in the intestinal microbiome could influence lung alveolar and vascular development and repair after oxygen exposure in a murine model of BPD. This is the first known study to suggest that hyperoxia alters the intestinal microbial composition over time when combined with antibiotic exposure. Our study also found that antibiotics cause long term changes in the composition of the intestinal microbiome. There seems to be a delayed effect of perinatal antibiotic exposure on the gut microbiome with a greater difference noted at PND 21 despite recovery in room air conditions. Furthermore, exposure to hyperoxia combined with antibiotic exposure leads to distinct gut microbiome changes compared to antibiotic exposure alone. However, we did not find any alterations in the lung microbiome after antibiotic exposure. This finding suggests that the intestinal microbiome as opposed to the lung microbiome may be most vulnerable to adverse exposures during the neonatal period.

Prior to our experiments with hyperoxia, we analyzed the changes in intestinal and lung microbial composition after antibiotic exposure with ampicillin only (perinatal antibiotic exposure was given to both dams and neonates). Even with a single antibiotic exposure, beta diversity was significantly altered in the neonatal intestinal microbiome at PND 6, and this persisted until PND 21. We also observed that ampicillin alone caused specific changes in the gut microbial composition over time, specifically, decreased relative abundance of *Bacteroides* and *Akkermansia* at PND 21. Our results are consistent with human studies that have found maternal intrapartum antibiotic prophylaxis (IAP) exposure to be associated with prolonged low levels of *Bacteroides* in infants^24^. *Bacteroides* are thought to be a beneficial gut bacteria and studies have suggested that low levels of *Bacteroides* may be associated with inflammatory bowel disease^25^. *Akkermansia* is also thought to be a beneficial bacteria in the human gut that resides in the mucin layer and a lower relative abundance may be associated with obesity^26^. Interestingly, we did not find any significant changes in alpha diversity measures, beta diversity, or microbial composition in the lung microbiome between ampicillin exposed neonatal pups and controls. This is consistent with other studies that have analyzed the neonatal murine lung microbiome and did not find changes after antibiotic exposure^19^.

In the current study, we also investigated the difference in intestinal microbiome diversity, richness and evenness between neonatal mice exposed to a three-antibiotic cocktail perinatally with or without oxygen exposure. It has been well established in both human and animal studies that maternal and neonatal antibiotic exposure alter gut microbiome diversity and microbial composition^12,18,19,24,27–30^. In our study, alpha diversity was minimally decreased at PND 21 in mice exposed to antibiotics, although this was not statistically different compared to non-exposed neonatal mice. This was consistent with Yassour *et al*. (2016) who saw a minimal decrease in alpha diversity after antibiotic exposure in a human infant study within the first three years of life suggesting a potential partial recovery after the exposure^28^. However, other human studies have found significant changes in species richness and diversity in the intestinal microbiome in neonates exposed to IAP^24,27^. Our minimal decrease in alpha diversity may be explained by the shorter time span of antibiotic delivery and administering antibiotics to dams and not directly to neonatal mice. This may have dampened the effect of antibiotics on the neonatal murine gut microbiome.

Although alpha diversity was not significantly modified in our study, we found that beta diversity or the difference in microbial composition between antibiotic exposed neonatal mice and controls was significantly altered at our PND 21 timepoint in both hyperoxia and room air groups. This suggests that the impact of antibiotic exposure on the gut microbiome can persist beyond the immediate newborn period and does not necessarily recover completely after a perinatal insult. The genera that were significantly decreased with three antibiotic exposure included *Bacteroidales*, *Romboutsia*, *Alistipes*, and *Parasutterella*. As opposed to ampicillin only, in the triple antibiotic exposed group, relative abundance of *Akkermansia* was increased. However, it is to be noted that pups were not directly administered antibiotics in the triple-antibiotic exposure model. This was in agreement with Gray *et al*. who found differences in intestinal beta diversity and alterations in the relative abundance of species between antibiotic exposed and non-exposed neonatal mice after maternal dams were exposed to antibiotics prenatally^19^. As in our study, a decrease in the phylum *Bacteroidetes* after maternal IAP has been shown in human studies as well^12^.

Interestingly, we found that when neonatal mice were exposed to hyperoxia in combination with antibiotics, there was a significant difference in beta diversity and the microbial composition compared to mice exposed to antibiotics and room air at PND 21. In addition, the original impact of the antibiotic exposure on certain bacteria appeared to be reversed after hyperoxia exposure. In the hyperoxia and antibiotic exposed cohort, the relative abundance of the genus *Akkermansia* was significantly suppressed after oxygen exposure although it had initially been able to flourish in the antibiotic only exposed mice. The genera *Bacteroidales* and *Alistipes* had a significantly higher relative abundance after oxygen and antibiotic exposure when compared to mice with just antibiotic exposure alone. These changes in the microbiome after hyperoxia exposure were only seen in those mice exposed to antibiotics and were not seen between hyperoxia and room air groups exposed to PBS. While many factors are known to alter the human microbiome, such as antibiotics, this study suggests that early oxygen exposure in combination with early antibiotics can also influence the neonatal microbiome. In the neonatal intensive care unit, this could have a significant impact on the preterm infant exposed to oxygen after birth due to respiratory distress syndrome, especially in the extremely low birth weight infants who often go on to develop BPD due to prolonged hyperoxia exposure. This finding proposes that alterations in the gut microbiome may be substantial in those infants with BPD due to their increased lifetime exposure to hyperoxia and antibiotics^1–3^.

This study analyzed the impact of antibiotic exposure on lung development and repair after hyperoxia exposure in neonatal mice. We did not find a significant difference in lung morphometry, specifically, alveolar simplification and vascular development between neonatal mice exposed to a three antibiotic cocktail and hyperoxia compared to hyperoxia alone. This is possibly due to the limited sample size and the particular mouse model of postnatal hyperoxia exposure used in our laboratory with a very short duration of oxygen exposure. Antibiotic exposure given only to maternal dams may also have dampened the impact of antibiotics on lung development and repair after hyperoxia. Other murine studies that have found connections between the gut microbiome and the lung used infection to investigate the impact of an altered gut microbiome on survival^18,19,29,30^. The intestinal microbiome is known to have a significant role in the developing neonatal immune system; therefore, infecting antibiotic exposed mice to elucidate the gut-lung axis would likely create a greater discrepancy between controls and experimental groups than oxygen exposure as in our study. However, we did find that hyperoxia significantly altered lung morphometry at PND 21 with significant alterations in mean linear intercept, radial alveolar counts, and small vessel counts when compared to room air controls. This is consistent with other studies that have used this model of lung injury to mimic human BPD^17^.

While our murine model of BPD produces a similar phenotype as human neonatal BPD, the use of 95% oxygen is not as common in the neonatal population in the post surfactant era. Our model targets the early alveolar stage of lung development when most human infants are vulnerable to BPD development. However, we only deliver a short course of hyperoxia which does not create as significant fibrosis, alveolar thickening, and inflammation as longer exposures might^31^. This shorter duration of hyperoxia exposure combined with a short course of antibiotics only given to maternal dams in our study may not have created enough intestinal dysbiosis and inflammatory response to impact the developing lung. Extremely preterm newborns typically receive multiple courses of antibiotics during their hospitalization, and these infants may also have other morbidities such as sepsis, necrotizing enterocolitis, and ventilator associated pneumonia that could potentially have an impact on the developing microbiome. Simulating a prolonged or repetitive antibiotic course or a multiple hit model in future murine studies may recapitulate the arrest in lung development seen in human neonates with BPD.

In conclusion, oxygen exposure may alter the intestinal microbiome in combination with other insults such as antibiotic treatment. Maternal pre- and post-natal antibiotic exposure can significantly modify the neonatal murine intestinal microbiome over a prolonged time period. Antibiotics do not appear to alter the neonatal murine lung microbiome and it is more likely that the intestinal microbiome itself influences health and disease in the lung.

## Supplementary information

Supplemental Data
