# Supplementary material for: Impact of Early Life Antibiotic Exposure and Neonatal Hyperoxia on the Murine Microbiome and Lung Injury
Source: Sci Rep. 2019 Oct 18;9:14992. doi: 10.1038/s41598-019-51506-0 (PMC6802223; doi:10.1038/s41598-019-51506-0)
Supplement: Supplementary file 1 — Supplemental Data [file 41598_2019_51506_MOESM1_ESM.pdf]

Supplemental Data:

Impact of Early Life Antibiotic Exposure and Neonatal Hyperoxia on the Murine Microbiome and Lung Injury

Melissa H. Althouse, Christopher Stewart, Weiwu Jiang, Bhagavatula Moorthy, Krithika Lingappan

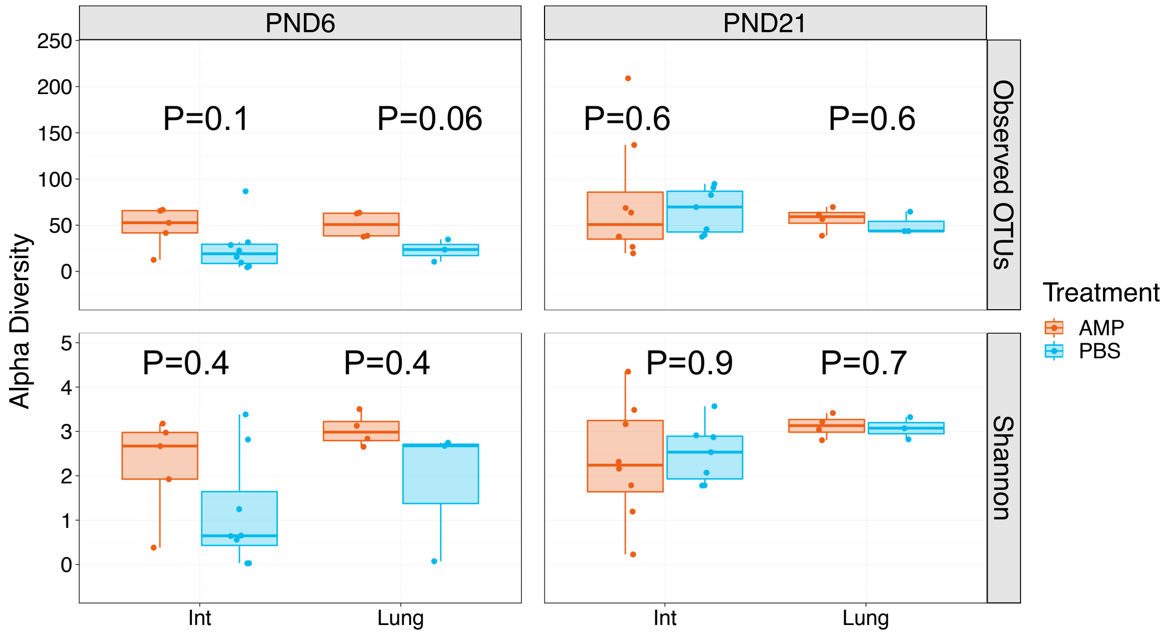

Supplemental Figure 1: Alpha diversity measures in the intestine and the lung at PND 6 and 21. Ampicillin treated mice did not show differences in Shannon Diversity or observed OTUs in the intestine or lung when compared to PBS treated controls at PND 6 or 21. AMP = ampicillin. Int = Intestine. PND = postnatal day. PBS = phosphate buffered saline.

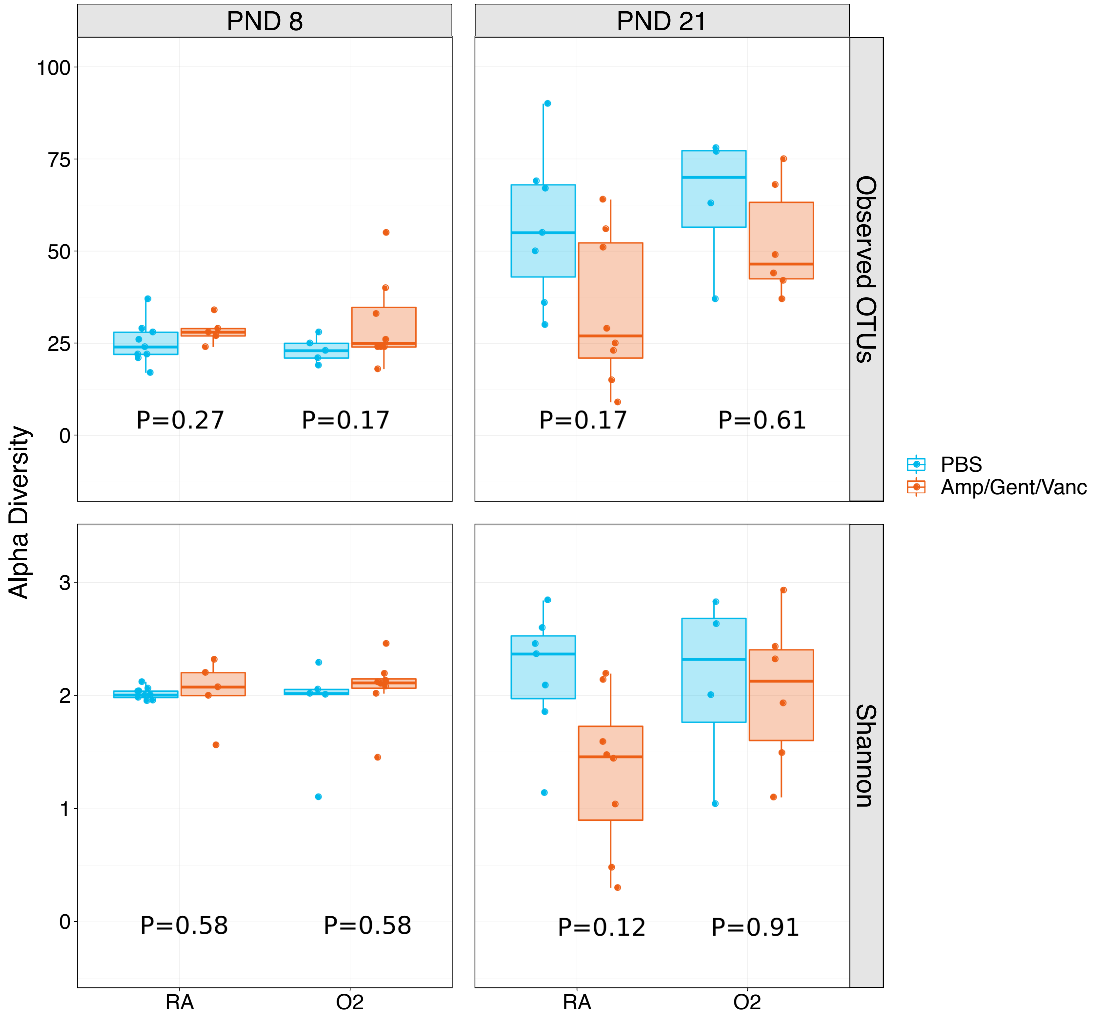

Supplemental Figure 2: Intestinal alpha diversity comparisons between three-antibiotic treated neonatal mice and controls at PND 8 and 21 in both room air and hyperoxia. Antibiotic treated mice did not show differences at PND 8 or PND 21 in either observed OTUs or Shannon Diversity when compared to PBS treated controls. RA = room air. O2 = hyperoxia. PBS= phosphate buffered saline. Amp/Gent/Vanc = ampicillin, vancomycin, gentamicin antibiotic cocktail.
